# Vision-related quality of life in patients treated for ocular syphilis

**DOI:** 10.1038/s41598-023-40289-0

**Published:** 2023-08-17

**Authors:** Milena Simões F. Silva, Tiago E. Arantes, Renata Moreto, Justine R. Smith, João M. Furtado

**Affiliations:** 1 Division of Ophthalmology, Ribeirão Preto Medical School, University of São Paulo, 3900 Bandeirantes Avenue, Ribeirão Preto, São Paulo 14049-900 Brazil; 2 Sadalla Amin Ghanem Eye Hospital, Joinville, Brazil; 3 Flinders University College of Medicine and Public Health, Adelaide, Australia

**Keywords:** Uveal diseases, Vision disorders

## Abstract

Multiple studies have showed negative impact of non-infectious uveitis on quality of life (QoL). Less is understood regarding life experiences in patients with infectious uveitis. We investigated vision-related QoL in individuals who had recovered from ocular syphilis. 32 adults treated for ocular syphilis at a uveitis service in Brazil completed the 25-item National Eye Institute Visual Function Questionnaire (NEI VFQ-25), and a comprehensive ophthalmic examination was performed. Medical records were reviewed to confirm resolution of ocular inflammation for 3 months pre-enrolment, and collect clinical data. The NEI VFQ-25 composite score was low overall (75.5 ± 19.8, mean ± standard deviation), and subscale scores varied from relative lows of 59.1 ± 39.6 (driving) and 60.9 ± 24.5 (mental health), to relative highs of 84.8 ± 21.8 (ocular) and 89.1 ± 21.0 (color vision). Adults aged over 40 years and those with a final visual acuity of 20/50 or worse had significantly lower mean composite and subscale scores. Other clinical characteristics—including gender, HIV co-infection, and type of uveitis—did not significantly influence scores. Our findings, taken in context with previous observations that prompt recognition achieves better vision outcomes, suggest early treatment may improve QoL after recovery from ocular syphilis.

## Introduction

Ocular syphilis has been re-emerging since approximately 2000, now accounting for more than 5% of patients referred to some tertiary referral inflammatory eye disease clinics^1^. The condition manifests as uveitis in the majority of patients, and while posterior and pan- uveitis are the most common forms, anterior and intermediate uveitis also occur^2–4^. Indeed, ocular syphilis has been nicknamed ‘the great masquerader’ due to the myriad of presentations that have been described^5^. Unlike most other forms of uveitis, the treatment of ocular syphilis is straightforward and inexpensive, and it is definitive: per the recommendations of the United States Centers for Disease Control and Prevention recommendations, a 10 to 14 day course of intravenous penicillin G, or ceftriaxone in the case of allergy, is curative^6^.

Taken as a group, uveitis causes 5% to 20% of blindness in industrialized countries and 25% in developing regions^7^, and approximately two-thirds of affected individuals experience prolonged vision loss^8^. Appropriately, there is an increasing interest in quality of life or patient-reported outcomes in uveitis. Most work has focused on non-infectious forms of uveitis, including groups of patients with different diagnoses from individual clinics^9–12^ or enrolled in randomized controlled trials^13,14^, as well as those with specific diagnoses, such as HLA-B27-asociated, Behçet disease and Vogt-Koyanagi-Harada disease^15–17^. These studies have demonstrated clear negative impact of the disease on quality of life, to an extent greater than that seen in diabetic retinopathy^18^ and age-related macular degeneration^19^.

A limited number of studies focused on certain subtypes—herpetic anterior uveitis and ocular toxoplasmosis^20–23^—suggest infectious uveitis also may influence a patient’s quality of life in some clinical settings. However, the effect of ocular syphilis on vision-related wellbeing and functioning has not been reported. In this study, we investigated quality of life in individuals who had been diagnosed with ocular syphilis and completed treatment at a tertiary referral uveitis clinic in Brazil, using a validated, interview-based Portuguese language version of the widely utilized visual function questionnaire developed by the US National Eye Institute^24^. Specifically, we aimed to identify clinical characteristics of ocular syphilis that were associated with reduced quality of life.

## Methods

### Identification of study participants

This cross-sectional study was conducted at the Uveitis Clinic of Ribeirão Preto General Hospital, representing the only tertiary uveitis service in the region of Ribeirão Preto, São Paulo, Brazil, and serving approximately 1.7 million citizens. The study was approved by the Ethics Committee of Human Research at Ribeirão Preto General Hospital (approval number: 57349616.1.0000.5440), and the research was performed in accordance with the Declaration of Helsinki.

Adults aged 18 years or older, who had been diagnosed with and treated for ocular syphilis between January 2000 and April 2019, were identified by a search of hospital medical records, and contacted by telephone call or approached during a clinic attendance with an invitation to participate in the study. Subjects were enrolled in the study, and interviewed and examined, between August 2016 and April 2019. Individuals who could not be contacted or did not agree to participate were not included, and written informed consent was obtained for all participants.

The diagnosis of ocular syphilis was based on a documented history of ocular inflammation that resolved following intravenous treatment with aqueous penicillin G or ceftriaxone, with serological confirmation of syphilis by a reactive treponemal (fluorescent treponemal antibody absorption, FTA-abs) test and a non-treponemal (venereal disease research laboratory, VDRL]) test with a titer 1:2 or higher.

### Collection of clinical data

The medical record was used to verify the diagnosis of ocular syphilis, and to confirm that the ocular inflammation had been inactive for at least 3 months prior to enrolment in the study. Additional data that were collected from the medical record included: self-identified gender, age at presentation with uveitis, human immunodeficiency virus (HIV) serology, type of ocular inflammation, type of uveitis, laterality of uveitis, presenting best-corrected visual acuity (BCVA), and treatment regimen. The type of uveitis was defined anatomically, using the Standardization of Uveitis Nomenclature (SUN) classification^25^. Isolated papillitis was classified as posterior uveitis for the purposes of the statistical analysis.

A comprehensive ophthalmic evaluation was performed, including measurement of BCVA, slit-lamp examination and indirect ophthalmoscopy with pupillary dilation. The BCVA of the better seeing eye was used for evaluating associations between clinical characteristics and vision-related quality of life, since this is strongly correlated with self-reported visual disability^26^. A BCVA of 20/50 or worse defined vision loss.

### Vision-related quality of life assessment

The 25-item National Eye Institute Visual Function Questionnaire (NEI VFQ-25) was used to assess the patient-perceived vision-related quality of life^24^. A validated Portuguese language interview format version was employed; this had 39 total items, including 14 additional items from the original 51-item NEI-VFQ^27^.

The NEI VFQ-25 generates 12 subscales: general health, general vision, ocular pain, near activities, distance activities, vision-specific social functioning, vision-specific mental health, vision-specific role difficulties, vision-specific dependency, driving, color vision, and peripheral vision. Higher scores indicate a better quality of life; lower scores indicate a worse quality of life. Following the standard instructions for analysis of the NEI VFQ-25, scores for each item were converted to a 0 to 100 scale, with the lowest and the highest possible scores set at 0 and 100 points, respectively. Items within each subscale were averaged to give 12 subscale scores. A composite score was calculated by averaging all subscale scores with the exception of the general health score.

### Statistical analysis

Statistical analysis was performed using Microsoft Excel 16.64 for Mac (Microsoft Corporation, Redmond, WA) and SPSS 16.0 for Windows (SPSS Incorporated, Chicago, IL). Continuous variables were expressed as the mean ± standard deviation (SD). Categorical variables were expressed as absolute and relative frequencies. Student’s t-test, Fisher’s exact test, Fisher-Freeman-Halton’s exact test, and Mann–Whitney U test were used to compare groups. A p-value of less than 0.05 was taken to indicate a statistically significant difference between groups.

## Results

Of a total of 50 eligible individuals, 32 patients (8 women including one transgender person, and 24 men, ranging in age from 23 to 80 years) participated in this study. Five patients (15.6%) were infected with HIV; CD4 counts were 317 to 994 cells/mm^3^ and viral loads were undetectable to 58.685 copies/ml in three subjects, and not available for two subjects. The most common diagnosis was posterior uveitis (n = 20 patients, 62.5%), followed by panuveitis (n = 11 patients, 34.4%) and intermediate uveitis (n = 1 patient, 3.1%). No patients had isolated anterior uveitis, or an ocular surface inflammatory disease including scleritis. Nineteen patients (59.4%) had bilateral uveitis: of the 51 affected eyes, there were 34 (66.%) with posterior uveitis (66.7%), 15 with panuveitis (29.4%) and 2 with intermediate uveitis (3.9%). More patients were treated with intravenous penicillin G than intravenous ceftriaxone (n = 19, 59.4% versus n = 13, 40.6%), and adjunctive corticosteroid treatment was commonly prescribed (n = 21, 65.6%).

Table 1 presents clinical characteristics of the study participants, and shows results of the analysis of vision outcomes. Across the 32 patients, initial BCVA in the better seeing eye was better than 20/50 in 19 (59.4%), 20/50–20/150 in 6 (18.7%), and 20/200 or worse in 7 (21.9%). Following treatment and after complete resolution of the ocular inflammation, BCVA in that eye of the patient was better than 20/50 in 26 (81.3%), 20/50–20/150 in 5 (15.6%), and 20/200 or worse in 1 (3.1%). None of the clinical characteristics that were recorded (including demographics, type of uveitis, presenting BCVA, and treatment) were significantly associated with the final BCVA in the better-seeing eye (p > 0.05).

**Table 1:** Clinical characteristics of patients with ocular syphilis (n = 32 individuals) subdivided by best-corrected visual acuity after treatment and resolution of disease.

Characteristic	All patients, n = 32	Final BCVA > 20/50, n = 26	Final BCVA ≤ 20/50, n = 6	*p*-value
Age at diagnosis, mean ± SD (years)	47.8 ± 16.1	45.4 ± 16.6	58.2 ± 8.5	0.079^b^
≤ 40 years	11 (34.4%)	11 (100.0%)	0 (0.0%)	0.071^c^
> 40 years	21 (65.6%)	15 (71.4%)	6 (28.6%)	
Gender, n (%)				
Woman	8 (25.0%)	7 (87.5%)	1 (12.5%)	> 0.999^c^
Man	24 (75.0%)	19 (79.2%)	5 (20.8%)	
HIV infection, n (%)				
Yes	5 (15.6%)	5 (100.0%)	0 (0.0%)	0.555^d^
No	27 (84.4%)	21 (77.8%)	6 (22.8%)	
Classification of uveitis, n (%)				
Posterior	20 (62.5%)	17 (85.0%)	3 (15.0%)	> 0.999^d^
Intermediate	1 (3.1%)	0 (0.0%)	1 (100.0%)	
Panuveitis	11 (34.4%)	9 (81.8%)	2 (18.2%)	
Initial BCVA, n (%)				
> 20/50	19 (59.4%)	17 (89.5%)	2 (10.5%)	0.194^c^
≤ 20/50	13 (40.6%)	9 (69.2%)	4 (30.8%)	
Antibiotic treatment, n (%)				
IV penicillin G	19 (59.4%)	13 (68.4%)	6 (31.6%)	0.059^c^
IV ceftriaxone	13 (40.6%)	13 (100.0%)	0 (0.0%)	
Corticosteroid treatment^a^, n (%)				
Yes	21 (65.6%)	18 (85.7%)	3 (14.3%)	0.390^c^
No	11 (34.4%)	8 (72.7%)	3 (27.3%)	

*SD* standard deviation, *HIV* human immunodeficiency virus, *BCVA* best-corrected visual acuity (in better seeing eye), *IV* intravenous.

^a^Corticosteroid treatment was oral prednisone in all cases, and one individual also was given a periocular triamcinolone acetonide injection prior to referral and diagnosis of ocular syphilis.

Statistical analyses were performed by: ^b^Student’s t-test, ^c^Fisher’s exact test, ^d^Fisher–Freeman–Halton exact test.

Mean composite score and subscale scores of the NEI VFQ-25 completed by the 32 patients are presented in Table 2. Taking the patients as a total, mean composite score was 75.5 ± 19.8, and mean scores for the 12 subscales varied from lows of 59.1 ± 39.6 (driving) and 60.9 ± 24.5 (mental health), to highs of 84.8 ± 21.8 (ocular) and 89.1 ± 21.0 (color vision).

**Table 2:** Subscale and composite scores on the National Eye Institute Visual Function Questionnaire-25 for patients with ocular syphilis (n = 32 individuals) presented as mean ± standard deviation overall and for different clinical characteristics.

Characteristic	Score												
	General health	General vision	Ocular pain	Near activities	Distance activities	Social functioning	Mental health	Role difficulties	Dependency	Driving	Color vision	Peripheral vision	Composite
Overall, n = 32 (100%)	66.7 ± 16.7	69.8 ± 20.0	84.8 ± 21.8	74.9 ± 23.1	75.2 ± 24.5	84.4 ± 24.9	60.9 ± 24.5	71.3 ± 29.3	82.8 ± 25.5	59.1 ± 39.6	89.1 ± 21.0	74.2 ± 27.3	75.5 ± 19.8
Gender, n (%)													
Woman, 8 (25%)	60.1 ± 18.2	72.5 ± 8.9	95.9 ± 26.3	71.8 ± 16.2	69.8 ± 20.3	81.2 ± 22.6	59.4 ± 27.8	75.0 ± 18.3	86.7 ± 16.2	56.7 ± 38.4	93.8 ± 17.7	75.0 ± 29.9	76.0 ± 15.9
Man, 24 (75%)	68.8 ± 16.2	69.0 ± 22.6	84.4 ± 20.6	76.0 ± 25.2	77.0 ± 25.8	85.4 ± 26.0	61.5 ± 23.9	70.1 ± 32.4	81.5 ± 28.1	59.7 ± 40.9	87.5 ± 22.1	73.9 ± 27.1	75.3 ± 21.2
Age, n (%)													
≤ 40 years, 11 (34%)	74.4 ± 16.1	78.2 ± 12.7	86.6 ± 12.3	**93.4 ± 6.2**	**91.4 ± 10.9**	**98.6 ± 3.2**	**76.8 ± 14.0**	**95.0 ± 7.8**	**98.9 ± 2.4**	**89.2 ± 11.8**	97.7 ± 7.5	**95.5 ± 10.1**	**91.0 ± 6.3**
> 40 year, 21 (66%)	62.7 ± 15.9	65.5 ± 22.0	83.9 ± 24.2	**65.4 ± 22.9**	**66.8 ± 25.5**	**77.1 ± 28.1**	**52.6 ± 24.9**	**58.9 ± 29.0**	**74.3 ± 28.1**	**35.9 ± 37.8**	84.5 ± 24.3	**63.1 ± 26.9**	**67.3 ± 19.6**
HIV infection, n (%)													
Yes, 5 (15.6%)	69.0 ± 21.7	65.0 ± 23.7	72.5 ± 16.3	79.2 ± 25.8	73.4 ± 22.6	81.7 ± 24.6	65.0 ± 20.3	75.0 ± 36.7	75.0 ± 39.8	68.8 ± 46.4	90.0 ± 22.4	75.0 ± 30.6	74.5 ± 23.5
No, 27 (84.4%)	66.3 ± 16.1	70.7 ± 19.6	87.0 ± 22.1	74.2 ± 23.0	75.6 ± 25.2	85.0 ± 25.4	60.2 ± 25.4	70.6 ± 28.6	84.3 ± 22.8	57.0 ± 39.1	88.9 ± 21.2	74.1 ± 27.3	75.6 ± 19.5
Bilateral involvement, n (%)													
Yes, 19 (59.4%)	62.1 ± 16.7	70.8 ± 18.2	78.8 ± 27.7	75.2 ± 21.8	76.9 ± 19.2	93.7 ± 7.7	63.1 ± 20.4	74.5 ± 22.7	85.5 ± 19.2	65.4 ± 35.2	88.5 ± 19.4	71.2 ± 22.5	77.1 ± 14.0
No, 13 (40.6%)	69.9 ± 16.5	69.2 ± 21.6	88.8 ± 16.1	74.8 ± 24.5	74.1 ± 28.0	78.2 ± 20.4	59.5 ± 27.4	69.1 ± 22.8	80.9 ± 29.4	56.3 ± 42.1	89.5 ± 22.5	76.3 ± 30.6	74.4 ± 23.2
Classification of uveitis^a^, n (%)													
Posterior, 20 (62.5%)	68.6 ± 19.1	67.5 ± 22.5	84.4 ± 24.3	73.5 ± 25.5	76.0 ± 26.6	87.9 ± 23.3	66.0 ± 22.4	74.7 ± 29.4	81.6 ± 29.6	66.7 ± 39.8	93.8 ± 17.9	73.8 ± 27.5	76.9 ± 20.8
Panuveitis, 11 (34.4%)	63.4 ± 12.8	76.4 ± 12.7	86.4 ± 18.1	79.9 ± 17.8	78.8 ± 14.0	84.8 ± 17.0	55.9 ± 24.8	69.3 ± 27.9	86.4 ± 17.6	53.1 ± 36.4	84.1 ± 23.1	79.6 ± 24.5	76.5 ± 14.8
Initial BCVA, n (%)													
> 20/50, 19 (59.4%)	67.5 ± 16.2	72.4 ± 16.5	82.9 ± 24.7	78.1 ± 19.9	**83.1 ± 19.0**	**95.2 ± 6.9**	**70.3 ± 17.7**	**81.9 ± 20.6**	91.8 ± 16.9	72.6 ± 34.5	92.1 ± 16.8	80.3 ± 24.4	81.9 ± 14.0
≤ 20/50, 13 (40.6%)	65.6 ± 18.1	66.1 ± 24.5	87.5 ± 16.9	70.4 ± 27.3	**63.8 ± 27.6**	**68.6 ± 32.8**	**47.3 ± 27.2**	**55.7 ± 33.8**	69.7 ± 30.6	37.9 ± 39.3	84.6 ± 26.1	65.4 ± 29.8	66.1 ± 23.5
Antibiotic treatment, n (%)													
IV penicillin G, 19 (59.5%)	66.4 ± 18.2	65.3 ± 23.2	83.6 ± 22.1	68.2 ± 26.5	69.0 ± 26.5	78.9 ± 29.4	57.4 ± 26.4	62.5 ± 33.2	77.3 ± 30.3	51.3 ± 44.1	85.5 ± 24.0	69.7 ± 30.7	70.3 ± 22.8
IV ceftriaxone, 13 (40.6%)	67.2 ± 15.0	76.5 ± 12.0	86.5 ± 21.9	84.8 ± 12.1	84.3 ± 18.3	92.3 ± 13.8	66.2 ± 21.3	84.2 ± 16.3	90.9 ± 13.7	69.2 ± 32.2	94.2 ± 15.0	80.8 ± 20.8	82.9 ± 11.3
Corticosteroid treatment, n (%)													
Yes, 21 (65.6%)	68.0 ± 12.5	72.9 ± 15.1	88.1 ± 15.6	77.7 ± 19.8	79.0 ± 20.7	86.2 ± 24.1	61.9 ± 22.8	74.1 ± 26.1	89.6 ± 16.2	58.8 ± 39.5	91.7 ± 18.3	75.0 ± 26.2	78.0 ± 16.6
No, 11 (34.4%)	64.4 ± 23.4	64.1 ± 27.0	78.4 ± 30.2	69.7 ± 28.6	68.0 ± 30.2	81.1 ± 27.4	59.1 ± 28.5	65.9 ± 35.4	69.8 ± 34.8	60.4 ± 45.8	84.1 ± 25.7	72.7 ± 30.5	70.6 ± 25.0
Final BCVA, n (%)													
> 20/50, 26 (81.3%)	68.2 ± 16.3	**74.2 ± 15.3**	85.1 ± 20.0	**81.0 ± 18.9**	**82.2 ± 18.6**	**89.4 ± 17.7**	**67.7 ± 20.5**	**80.8 ± 21.6**	**89.7 ± 20.4**	67.9 ± 34.3	93.3 ± 16.7	78.9 ± 24.2	**81.2 ± 15.2**
≤ 20/50, 6 (18.7%)	60.4 ± 18.9	**50.8 ± 28.0**	83.3 ± 30.3	**48.6 ± 22.0**	**45.0 ± 25.0**	**62.5 ± 39.7**	**31.7 ± 18.9**	**30.0 ± 22.5**	**53.1 ± 25.5**	0.0 ± 0.0	70.8 ± 29.2	54.1 ± 33.2	**50.5 ± 18.7**

*HIV* human immunodeficiency virus, *BCVA* best-corrected visual acuity, *IV* intravenous.

Statistical analyses were performed by Mann–Whitney U test, with bold values denoting statistical significance (*p* < 0.05).

^a^Intermediate uveitis was not included as this group included one individual.

**Figure 1:**
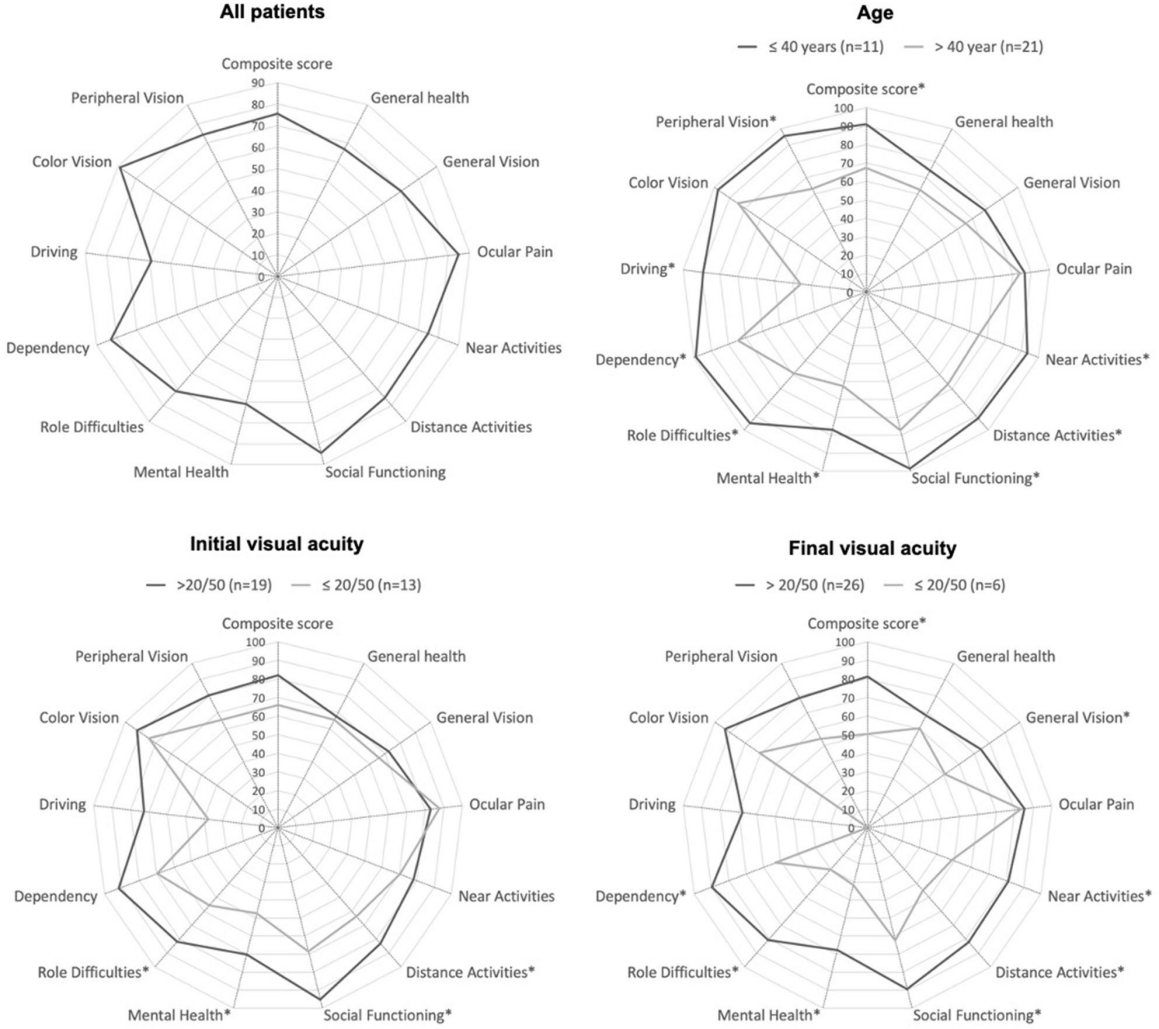
Mean subscale and composite scores on the National Eye Institute Visual Function Questionnaire-25 for patients with ocular syphilis (n = 32 individuals) overall and by age, and initial (presenting) and final (post-resolution) best-corrected visual acuities. Plots were created in Microsoft Excel 16.64 for Mac.

In comparison to patients with good final BCVA, those with a final BCVA of 20/50 or worse had significantly lower mean scores for general vision, near activities, distance activities, social functioning, mental health, role difficulties, and dependency subscales, as well as a significantly lower mean composite score (p < 0.05). For a subset of these subscales (distance activities, social functioning, mental health, and role difficulties), lower mean scores were also significantly associated with an initial BCVA of 20/50 or worse. In comparison to younger adults (40 years and less), middle and older aged adults (over 40 years) had significantly reduced scores for near activities, distance activities, social functioning, mental health, role difficulties, dependency, driving, and peripheral vision subscales, plus a significantly lower mean composite score (p < 0.05). Other clinical characteristics (including demographics, type of uveitis, and treatment) did not significantly influence mean composite score or subscale scores (p > 0.05). The differences in scores overall and by age, and initial and final BCVA are illustrated in Fig. 1.

## Discussion

Non-infectious uveitis has been shown to reduce an individual’s quality of life substantially^9–17^. There is less clarity around infectious uveitis: three studies of ocular toxoplasmosis drew different conclusions^21–23^, and a study of herpetic anterior uveitis showed a modest effect^20^. We assessed quality of life in a group of 32 patients who had recovered from ocular syphilis—manifested as intermediate, posterior or pan-uveitis—following standard treatment with antibiotics, supplemented with corticosteroid in approximately two-thirds. We found that middle and older aged adults, over 40 years when diagnosed and treated, experienced a lower overall quality of life than younger adults. Persons with reduced BCVA, reading 20/50 or worse after resolution of the uveitis, also reported reduced composite quality of life in comparison to those without vision loss.

Ocular syphilis has a readily available and inexpensive, safe and effective treatment that cures the condition^5^. On that basis, one might expect no impact on quality of life following treatment. However, our patients had a low mean composite scores (75.5 ± 19.8) when compared with the reference group in the original description of the NEI VFQ-25 (93.0)^24^, as well as groups of middle-aged and older Latinos without visual impairment (86.3 for English-speaking, 85.1 for Spanish-speaking)^28^. However, there may be a delay in reaching the diagnosis of ocular syphilis, and this has been cited as a reasons for poor outcome^29^. Indeed, visual recovery is incomplete in up to one-third of eyes, and a range of complications—cataract, glaucoma, macular pucker, optic atrophy and rhegmatogenous retinal detachment—may result in long-term visual disability^3,4,30^.

We found associations between quality of life scores across seven subscales (including general vision, near activities, and distance activities) in addition to the composite score for BCVA after resolution, and across four subscales (also including general vision, near activities, and distance activities), but not the composite for presenting BCVA. Patients with initial and final BCVA of 20/50 or worse had lower scores regardless of uveitis type and treatment. The same association between BCVA and scores on the NEI VFQ-25 has been reported in studies of other types of uveitis^13,19,31^. It was also noted during development of the questionnaire, with similar correlation for better and worse seeing eyes, especially in subscales related to general vision, near vision, and distance vision^24^.

In patients with ocular syphilis, being aged over 40 years was statistically correlated with lower quality of life. A large cross-sectional study from Germany that included 619 working adults showed a clear age dependency of NEI VFQ-25 scores: the composite score decreased by approximately one point for every decade of life, for adults with and without eye diseases^32^. Lower near and distance activity subscales of visual functioning were observed in patients with Behçet uveitis aged 30 years or older^16^; and general vision, near activities, and role difficulties subscales were negatively correlated an age of 45 years or more in herpetic anterior uveitis^20^. However, the opposite result of no correlation between age and NEI VFQ-25-derived quality of life scores have also been reported for groups of patients with non-infectious uveitis^11,12,18^ and ocular toxoplasmosis^22^.

Limitations of our study include the collection of data at a tertiary referral center, and the inability to recruit all eligible patients, potentially introducing selection bias. Also, discrepancies in sample size may have influenced some results: although we did not observe associations between quality of life, and gender, HIV infection status and type of uveitis, less than 20% of our patients were HIV-positive, 25% were women, and the majority had posterior uveitis. We did not attempt to correlate quality of life scores with ocular complications given the small size of the patient group. Despite these limitations, our study provides unique data on the quality of life in patients who have suffered from ocular syphilis. Given the association we observed between BCVA and NEI VFQ-25 scores, plus previous observations that prompt recognition achieves better visual outcomes^3,33,34^, we speculate that early diagnosis and treatment may result in higher quality of life for individuals with ocular syphilis.

## Data Availability

All data generated or analyzed during this study are included in this published article (and its Supplementary Information files).
